# Study of the pharmacokinetic changes of Tramadol in diabetic rats

**DOI:** 10.1186/2008-2231-21-17

**Published:** 2013-03-07

**Authors:** Hoda Lavasani, Behjat Sheikholeslami, Yalda H Ardakani, Mohammad Abdollahi, Lida Hakemi, Mohammad-Reza Rouini

**Affiliations:** 1 Biopharmaceutics and Pharmacokinetics Division, Department of Pharmaceutics, Faculty of Pharmacy, Tehran University of Medical Sciences, Tehran, 14155-6451, Iran; 2 Department of Pharmacology and Toxicology, Faculty of Pharmacy, Tehran University of Medical Sciences, Tehran, 14155-6451, Iran; 3 Pharmaceutical Sciences Research Centre, Faculty of Pharmacy, Tehran University of Medical Sciences, Tehran, 14155-6451, Iran

## Abstract

**Background:**

Besides the pathological states, diabetes mellitus may also alter the hepatic biotransformation of pharmaceutical agents. It is advantageous to understand the effect of diabetes on the pharmacokinetic of drugs. The objective of this study was to define the pharmacokinetic changes of tramadol and its main metabolites after *in vivo* intraperitoneal administration and *ex vivo* perfused liver study in diabetic rat model.

Tramadol (10 mg/kg) was administered to rats (diabetic and control groups of six) intraperitoneally and blood samples were collected at different time points up to 300 min. In a parallel study, isolated liver perfusion was done (in diabetic and control rats) by Krebs-Henseleit buffer (containing 500 ng/ml tramadol). Perfusate samples were collected at 10 min intervals up to 180 min. Concentration of tramadol and its metabolites were determined by HPLC.

**Results:**

Tramadol reached higher concentrations after i.p. injection in diabetics (C_max_ of 1607.5 ± 335.9 ng/ml) compared with control group (C_max_ of 561.6 ± 111.4). M1 plasma concentrations were also higher in diabetic rats compared with control group. M2 showed also higher concentrations in diabetic rats. Comparing the concentration levels of M1 in diabetic and control perfused livers, showed that in contrast to intact animals, the metabolic ratios of M1 and M5 (M/T) were significantly higher in diabetic perfused liver compared to those of control group.

**Conclusions:**

The pharmacokinetic of tramadol and its three metabolites are influenced by diabetes. As far as M1 is produced by Cyp2D6, its higher concentration in diabetic rats could be a result of induction in Cyp2D6 activity, while higher concentrations of tramadol can be explained by lower volume of distribution.

## Introduction

The capacity of organisms to eliminate xenobiotics such as pharmaceutical drugs and environmental pollutants from their body is subject to change. One of the best-known effective factors is the genetic variation of drug metabolizing enzymes and transporters. Numerous genetic polymorphisms have been reported with cytochrome P450 (P450s) [1]. In addition to the genetic background, xenobiotic-induced transcriptional activation or deactivation (i.e. induction or inhibition) has been documented in large numbers and drawing many researchers’ attention in order to avoid unfavorable drug–drug interactions and side effects of therapeutic drugs [2]. Physiological and pathophysiological conditions also affect the activity of P450s and other enzymes. Obesity and diabetes are worldwide concerns as risk factors for metabolic syndromes in the liver [3].

Diabetes mellitus, a disease with wide prevalence in humans, involves many complications including micro- and macro angiopathy as well as neuropathy, which in turn leads to increase the incidence of many diseases. Besides these pathological states, it is believed that possible diabetes-induced alterations in the hepatic biotransformation of pharmaceutical agents could also pose additional health risk because of dangerous side effects due to drug toxicity [4]. Considering the number of diabetic patients and their increased opportunities for drug therapy compared to healthy subjects, it is of great interest to understand the effect of this disease on drug metabolism.

Several chemicals have been used for induction of insulin-dependent diabetes mellitus in animal models, principally alloxan, streptozotocin and zinc chelators [5]. According to the literature, streptozotocin causes structural alterations in pancreatic beta cells (total degranulation) within 48 h after administration and last up to 4 months [5].

In rat models of diabetes mellitus induced by streptozotocin (DMIS) some physiological changes including a decrease in bile flow rate [6], hepatotoxicity [7], impaired renal function [8,9], disorders of the gastrointestinal tract and reductions in protein binding of drugs due to elevated plasma fatty acid level and/or glycosylation of plasma proteins [9], have been reported. Glucuronidation and sulfation were also strongly affected in DMIS rats [10]. Recently, it was shown that the expression of CYP1A1, 2A1, 2B1, 2C12, 2E1, 3A4, 4A1 and/or 4A2 were apparently increased in DMIS rats [11-14]. However, CYP2C11, 2C13, 2A2 and 3A2 were suppressed [12].

Tramadol hydrochloride (T) is a centrally acting analgesic with efficacy and potency ranging between weak opioids and morphine. The drug is mostly eliminated via biotransformation in the liver in two main pathways including *O*-demethylation to *O*-desmethyltramadol (M1) (the pharmacologically active metabolite) by isoenzyme cytochrome P450 2D6 (CYP2D6) and *N*-demethylation to *N*-desmethyltramadol (M2) by cytochromes P450 2B6 (CYP2B6) and 3A4 (CYP3A4) [15]. These primary metabolites may be further metabolized to three additional secondary metabolites namely, *N*, *N*-didemethyltramadol (M3), *N*, *N*, *O*-tridesmethyltramadol (M4) and *N*, *O*-desmethyltramadol (M5). The *O*-desmethylated metabolites are then further conjugated with glucuronic acid and sulfate before excretion in urine [15].

Recently, tramadol was suggested as an effective oral medication to alleviate pain in diabetic painful neuropathy (DPN) [16,17] and the dose-dependent lowering effect of tramadol on the plasma glucose levels of DMIS rats was also reported by Cheng et al. [18]. Although tramadol is known to be effective for the symptomatic relief of DPN, little definitive data is available concerning the effects of diabetes on hepatic drug metabolism and pharmacokinetics of this compound. Moreover, the results of those studies are not equivocal and are often contradictory.

The objective of this study was to investigate the pharmacokinetic changes of tramadol and its main metabolites after *in vivo* intraperitoneal administration and *ex vivo* perfused liver study in the DMIS rat model.

## Materials and methods

### Materials

**Figure 1:**
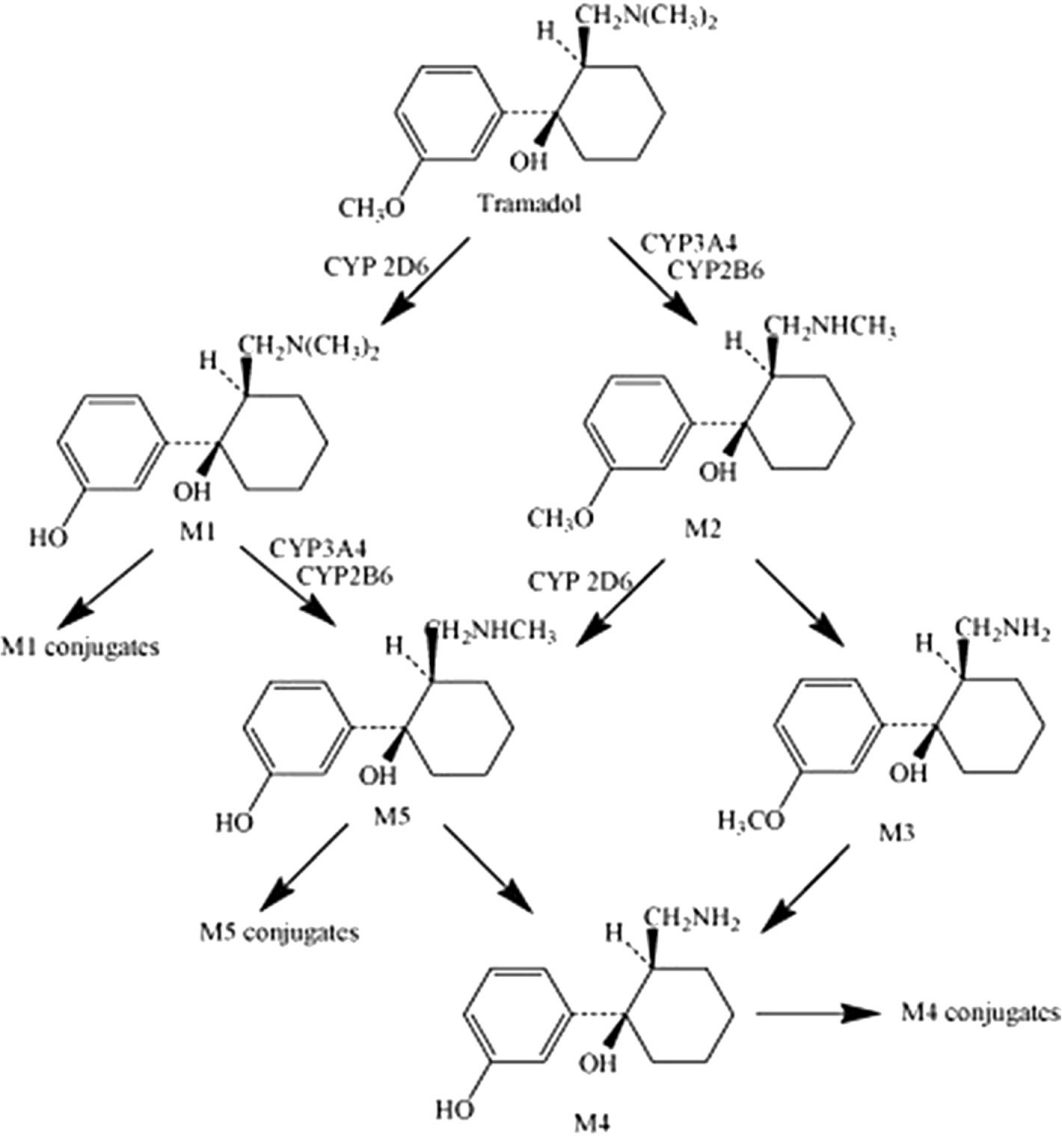
Metabolic pathway of tramadol.

The pure substances of tramadol, M1, M2, M5 and cis-tramadol as internal standard (Figure 1) were kindly supplied by Grǖnenthal (Achen, Germany). All other chemicals were supplied by Merck (Darmstadt, Germany). Water used in all experiments was of Direct-Q® quality (Millipore, France).

### Animals

Male Sprague–Dawley rats of 7 weeks old (weighing 250–300 g) were maintained in a clean room with 12 h light–dark cycle, controlled temperature environment between 20 and 23°C, a relative humidity of 50% and free access to standard laboratory chow and water. The study was approved by the Institutional Review Board of Pharmaceutical Research Centre of Tehran University of Medical Sciences. The animals were randomly divided into two experimental groups including control and DMIS rats.

### Induction of diabetes

The animals were made diabetic with a single intravenous injection of streptozotocin (Sigma, USA). Freshly prepared streptozotocin (60 mg/kg) in 0.9% saline containing 0.01 M sodium citrate (pH adjusted to 4.5) was administered once to the overnight- fasted rats via the tail vein [19]. An equal volume of a citrate buffer of pH 4.5 (0.3 ml) was injected to the control rats. On day 7 after intravenous administration of streptozotocin (rat models of DMIS) or a citrate buffer (controls for rat model of DMIS), non-fasting blood glucose levels of rats were measured using the Accu-Chek Active® (Roche Diagnostics, Basel, Switzerland). The diabetic state was confirmed by glucose levels exceeding 200 mg/dl.

### Intact diabetic rat study

At seventh day after the beginning of treatment with streptozotocin (DMIS rats), the femoral vein of one leg of DMIS and control rats were cannulated with previously heparinized intravenous 16–18 gauge catheter while each rat was under ketamine anesthesia using an intraperitoneal injection of xylazine/ketamine (15/75 mg/kg). Tramadol was administered intraperitoneally as 10 mg/kg, diluted in normal saline. The animals were kept under anesthesia until the end of the experiment. Approximately, 200 μl of venous blood samples were collected in heparinized tubes at: 0 (blank); 7.5, 15, 30, 45, 60, 90, 120, 165, 210, 255 and 300 min after tramadol administration. Blood samples were immediately centrifuged for 20 min at 1800 g and the plasma samples were stored at -80°C until HPLC analysis. A 250 μl of heparinized normal saline (15 units/ml) was used to flush the cannula to prevent blood clotting. At the end of the study, animals were sacrificed by cervical dislocation.

### Isolated liver perfusion study

The isolated liver perfusion study was also conducted in the DMIS and control rats. Animals were anesthetized using an intraperitoneal injection of xylazine/ketamine (15/75 mg/kg). The portal vein and superior vena cava were catheterized with an intravenous 16–18 gauge catheter, respectively. 500 units of heparin were injected into the inferior vena cava. Freshly prepared Krebs-Henseleit buffer (118 mm NaCl, 4.5 mm KCl, 2.75 mm CaCl2, 1.19 mm KH2PO4, 1.18 mm MgSO4 and 25 mm NaHCO3, equilibrated with 95% O2/ 5% CO2, pH 7.4) (containing 500 ng/ml tramadol) was passed through the portal vein with a constant flow rate of 10 ml/min using a peristaltic pump. By this method, perfusion medium passed through the liver and then collected from the superior vena cava [20]. The total volume of the reservoir was 200 ml. The temperature (37°C), pH (7.4) and perfusion pressure (14 mmHg) were periodically monitored and kept unchanged through the study. Liver variability was proved by monitoring the liver enzymes activities (AST and ALT). Perfusate samples were collected at 10 min intervals up to 180 min.

### Analytical method

Tramadol, M1, M2 and M5 concentration in rat plasma samples were determined by a previously described HPLC method [21]. Briefly, all analytes were extracted with ethylacetate and injected to a Knauer high-performance liquid chromatography (Berlin, Germany), equipped with a low-pressure gradient HPLC pump, a fluorescence detector, a Rheodyne injector with a 100 μL loop and an online degasser. Excitation and emission wavelength were 200 nm and 301 nm respectively. Separation was achieved by a Chromolith™ Performance RP-18e 100 × 4.6 mm column (Merck, Darmastadt, Germany) protected by a Chromolith™ guard cartridge RP-18e 5 × 4.6 mm. A methanol: water (adjusted to pH of 2.5 by phosphoric acid) mixture (19:81, v/v) at flow rate of 2 ml/min was used as mobile phase. Data acquisition was carried out by using ChromGate chromatography software (Knauer, Berlin, Germany).

### Pharmacokinetic analysis

The pharmacokinetics of tramadol and its metabolites were determined by non-compartmental analysis using Microsoft® EXCEL under Windows XP in both intact animal and isolated rat liver studies. Maximum plasma and perfusate concentrations (C_max_) of analytes and their corresponding times (Tmax) were recorded as observed. Elimination rate constant (β) was estimated as the absolute value of the slope of least-square linear regression of the terminal phase of the logarithmic concentration– time curves. The terminal half-life (t_1/2_) was calculated as 0.693/β. The area under the concentration versus time curve was calculated by the trapezoidal rule for the duration of sampling or last quantifiable concentration and extrapolated from the last point to infinity with β. Plasma clearance for tramadol (CL/F) was calculated as Dose/AUC_0–∞_. Metabolic ratios for AUCs or concentrations were calculated by dividing the AUC_0–t_ or concentrations of the metabolite by that of tramadol for plasma and perfusate samples.

### Statistical evaluation

Data was expressed as mean ± SD. To compare the pharmacokinetic parameters of tramadol and its metabolites in DMIS rats and control group, an unpaired t-test was used for all parameters except T*max*, with which a nonparametric Wilcoxon two-sample test was used. A p-value of less than 0.05 was considered to be statistically significant.

## Results and discussion

Studies showed that pharmacokinetic parameters of drugs can change by diabetes mellitus [22,23]. It has been suggested that plasma protein binding of drugs may change because of change in plasma fatty acid levels [9]. Moreover, an intracellular dehydration has been observed in male Sprague-Dawley DMIS rats [24]. Consequently both may affect the distribution of drugs in the body.

It has also been reported that cardiac index and the blood flow rate to the diaphragm, abdominal wall and kidney elevate in male Sprague-Dawley DMIS and Carworth Farms E (CFE) rats [25,26]. An increase in most cytochrome p450 isoenzymes activity has also been reported resulting in elevation of metabolites levels in diabetic rats [27].

Tramadol is quickly and almost entirely absorbed after an oral administration in human whereas its mean absolute bioavailability is reported to be only 65–70% as a result of the first-pass hepatic metabolism. The high total distribution volume of around 300 L after oral administration in human is caused by its high tissue affinity and distribution in fat tissues [28]. An enormous amount of tramadol is rapidly metabolized to three main metabolites in Liver. The principal metabolic pathways, *O*- and *N*-desmethylation, involve cytochrome P-450 isoenzyme 2D6, 2B6 and 3A4, respectively. The primary metabolites *O*-desmethyltramadol (M1) and *N*-desmethyltramadol (M2) may be further metabolized to *N*, *O*-didesmethyltramadol (M5). Ten to thirty percent of the parent drug is excreted unchanged in the urine. Tramadol and its metabolites are almost completely excreted via the kidneys and their biliary excretion is negligible [29].

Animal studies for diabetes especially in rats and mice have been commonly applied streptozocin to provide type 1 diabetes. It has been reported that intravenous administration of a dose ranging from 25 to 100 mg/kg STZ could successfully induce a dose dependent hyperglycemia in rats [30]. In this study, we used a single 60 mg/kg dose of STZ intravenously in order to induce the diabetes in rats.

### Intact animals

**Figure 2:**
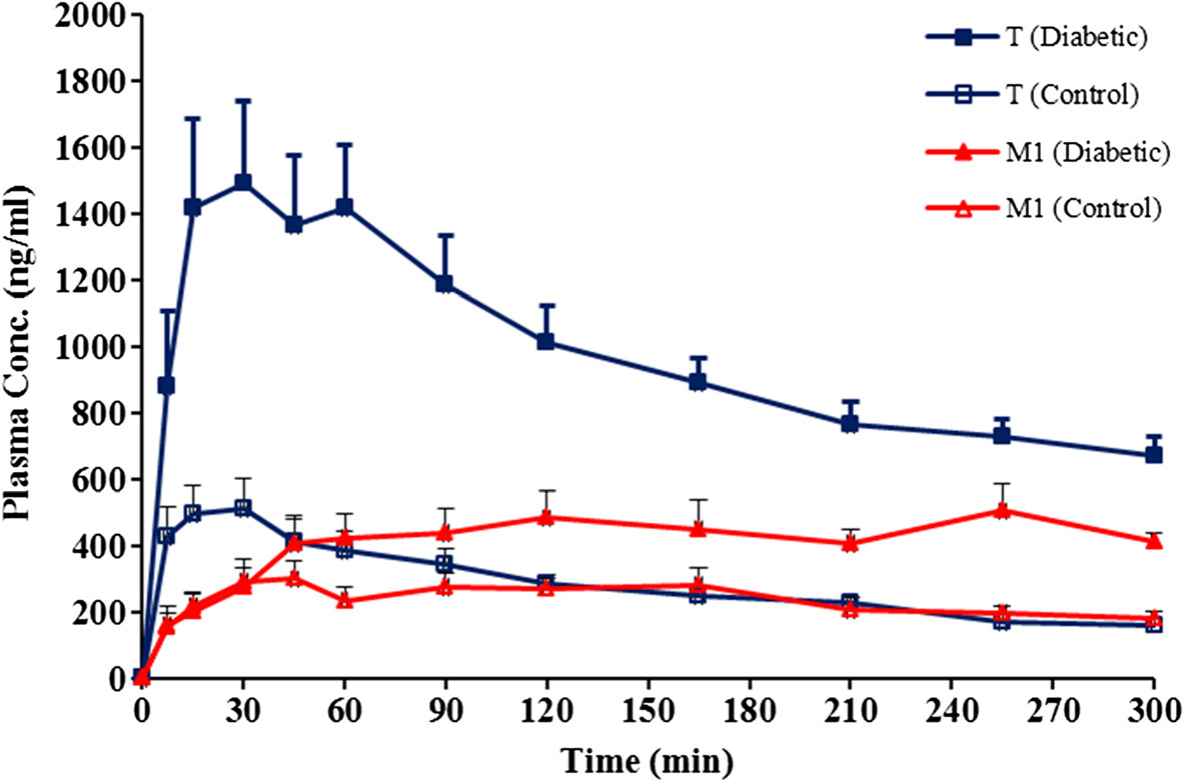
Plasma concentration-time profile of tramadol and M1 in intact control and diabetic rats after receiving a 10 mg/kg of tramadol intraperitoneally (n = 6 in each group, data are presented as mean ± SE).

**Figure 3:**
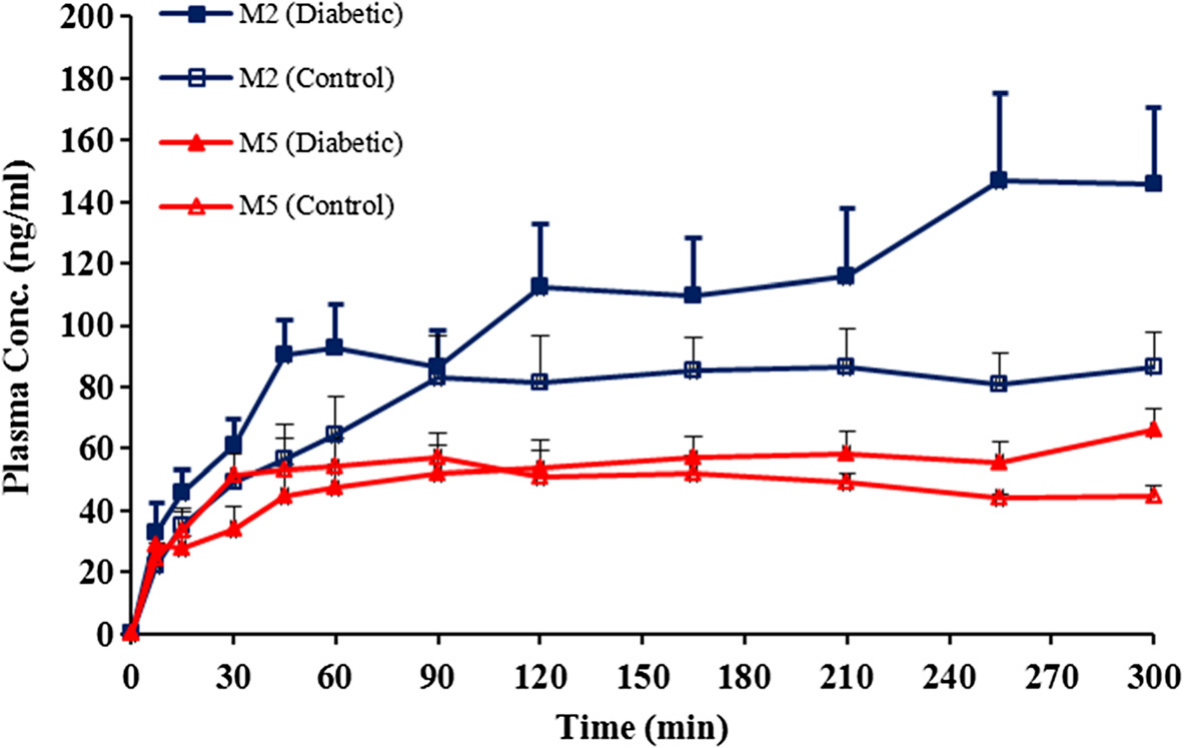
Plasma concentration-time profile of M2 and M5 in intact control and diabetic rats after receiving a 10 mg/kg of tramadol intraperitoneally (n = 6 in each group, data are presented as mean ± SE).

Six rats completed the study in each group. Mean body weight of rats in two study groups were not statistically different at the beginning (285 ± 12 vs. 295 ± 16 grams in control and treatment groups respectively, p > 0.05). While rats in treatment group showed a significant decrease in their body weight during 7 days of diabetes induction (295 ± 16 vs. 235 ± 11 grams respectively, p < 0.05), no significant changes was observed in body weight of control group animals during this period. Plasma glucose concentrations measured 7 days from the beginning of the study were significantly increased in animals given streptozotocin, 388.4 ± 164.4 mgdL^-1^ compared with the controls, 102.8 ± 31.1 mgdL^-1^ (P < 0.05). Animal in treatment group had much higher water consumption in comparison to control group. Plasma concentration-time profiles of tramadol and M1 are presented in Figure 2 and those of M2 and M5 in Figure 3 respectively. Calculated pharmacokinetic parameters for Tramadol and metabolites are presented in Tables 1 and 2 respectively.

**Table 1:** Pharmacokinetic parameters of tramadol in diabetic and control rats

**Parameter**	**AUC **_**0-300min **_**(ng.min/ml)**		**AUC **_**0-∞ **_**(ng.min/ml)**		**K**_**el **_**(1/min)**		**App. Clearance (ml/min)**	
	**Control**	**Diabetic**	**Control**	**Diabetic**	**Control**	**Diabetic**	**Control**	**Diabetic**
Mean	**88879.2**	**292661.1**	**133344.4**	**548924.4**	**0.0036**	**0.0026**	**22.3**	**5.5**
P Value	**<0.05**		**<0.05**		**>0.05**		**<0.05**	

**Table 2:** **AUC **_**0-300min **_**(ng.min/ml) of tramadol metabolites; M1, M2, M5**

**Parameter**	**M1**		**M2**		**M5**	
	**Control**	**Diabetic**	**Control**	**Diabetic**	**Control**	**Diabetic**
Mean	**70835.2**	**124988.4**	**22447.3**	**31947.2**	**14486.2**	**15505.6**
P Value	**<0.05**		**>0.05**		**>0.05**	

After i.p. injection in intact rats, tramadol was absorbed rapidly in both groups and reached much higher concentrations in diabetic in comparison to control group with a C_max_ of 561.6 ± 111.4 and 1607.5 ± 335.9 ng/ml in control and diabetic rats respectively (p < 0.05). However the time to C_max_ (T_max_) was longer in diabetics compared to control group (36.1 ± 17.1 min vs*.* 18.2 ± 11.4 min in diabetic and control rats respectively). Much higher area under plasma-concentration-time curve was also observed in diabetics in comparison to control group (292661.1 ± 49048.2 vs. 88879.2 ± 14483.4 ng.min.ml^-1^ respectively, p < 0.05). The terminal phase of tramadol plasma concentration-time profile in both groups showed no significant difference in elimination rate constant (0.0036 ± 0.0009 min^-1^ vs. 0.0026 ± 0.0008 min^-1^) in control and diabetic groups respectively, (p > 0.05).

It has been assumed that tramadol is metabolized much more rapidly in animals than in humans, and M1, M2 and M5 are the main metabolites in all species [31]. Our study also confirms that M1, M2 and M5 are formed and M1 remains the major metabolite in rat. However it is not clear if the same enzymes in human and rat are responsible for metabolite formation in these two spices. In the present study, M1 remained the major metabolite of tramadol in both diabetic and non-diabetic intact rats.

Similar to tramadol, the M1 plasma concentrations were higher in diabetic rats compared to control group which resulted in significantly higher AUCs in diabetics (124988.4 ± 33887.3 vs. 70835.2 ± 14341.5 ng.min.ml^-1^ in diabetic and control respectively). In both groups the concentration of M1 reached to an almost constant value in about 90 minutes after drug administration.

Similar to tramadol and M1, M2 showed higher concentrations in diabetics in comparison with control rats. The concentration of M2 in DMIS and control rats was increased up to last sampling point. However the increment trend in M2 concentration was much higher in diabetics resulted in marked increase in AUC of this metabolite in diabetics. As mentioned, diabetic group showed higher concentrations (almost 1.7 times in diabetic group after 300 min compared to control groups) and higher AUCs (31947.2 ± 9923.4 vs. 22447.3 ± 4871.6 ng.min.ml^-1^) compared to control group. In contrast to M2 and similar to M1, the M5 concentrations reached to a plateau in both groups almost 60 minutes after drug administration and there was no significant differences between AUC of both groups (15505.6 ± 2433.2 vs. 14486.2 ± 2119.4 in diabetic and control respectively) (p > 0.05).

**Figure 4:**
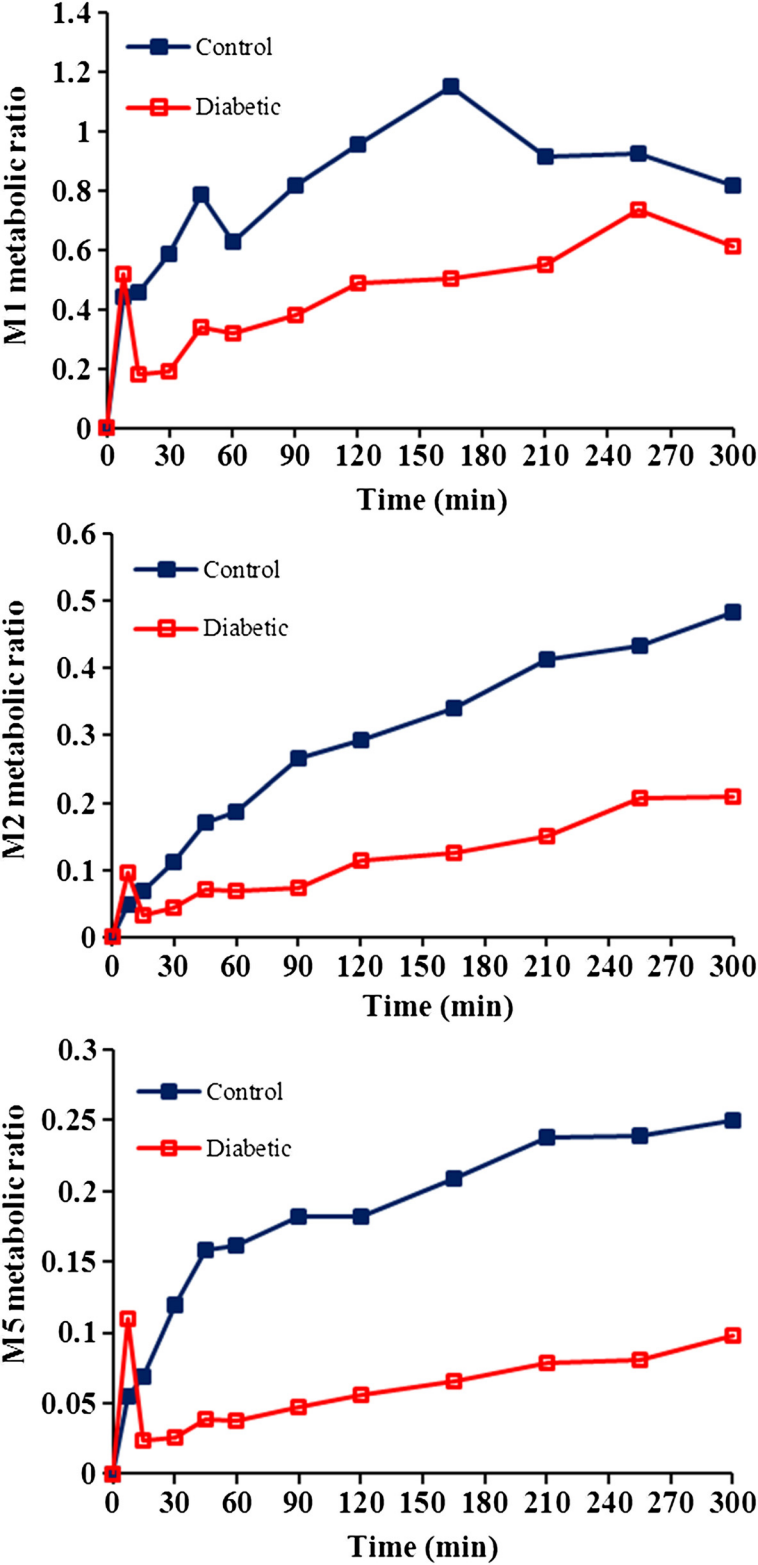
The metabolic ratio for M1, M2 and M5 in intact control and diabetic rats.

As it is clear from above explained data, the diabetes has markedly influenced pharmacokinetic of tramadol and its metabolites in rats. Figures 2 and 3 show that tramadol, M1 and M2 have higher concentrations in diabetic compared to control rats. Considering M1 as major metabolite and induction of its formation in diabetic rats, a reduction in plasma levels and AUC of tramadol might be expected. Table 1 show that contrary to our expectation, both AUC_0-300min_ and AUC_0-∞_ of tramadol are significantly higher in diabetic rats. To further pursue this argument, the metabolic ratios were studied for all three metabolites. Figure 4 shows that for all three metabolites, the metabolic ratio is higher in control in comparison to diabetic rats. These higher metabolic ratios in control rats may be caused by higher plasma levels of metabolites or lower tramadol plasma levels in control group. Lower plasma levels of tramadol could be resulted either from higher metabolism rate (contrary to our expectation) or higher distribution volume of the drug in control rats.

Diabetes may change the pattern of distribution of drugs in the body. Tramadol has a high volume of distribution in man [28]. Any reduction in fat tissue (which usually happens during diabetic state) may cause a reduction in volume of distribution of lipophilic drugs such as tramadol. Considering higher lipophilicity of tramadol compared to its metabolites and consequently higher distribution in fat tissue, such a phenomenon may also explain higher plasma concentration of tramadol in diabetic rats. In addition, the higher production level of alph1-acid glycoprotein, an acute-phase serum protein which is prominent in tramadol protein binding, in diabetic condition may further cause in decrease of volume of distribution and increase in plasma concentration of tramadol in diabetic rats. In isolated liver perfusion study, the metabolism of drugs could be investigated when to a high extent the effect of drug binding and distribution volume has been excluded. To further study the metabolic state of tramadol and clear the possible effect of distribution volume and protein binding on metabolite formation and metabolic ratios, an isolated rat liver study was performed in both normal and diabetic rat liver.

### Isolated liver study

**Figure 5:**
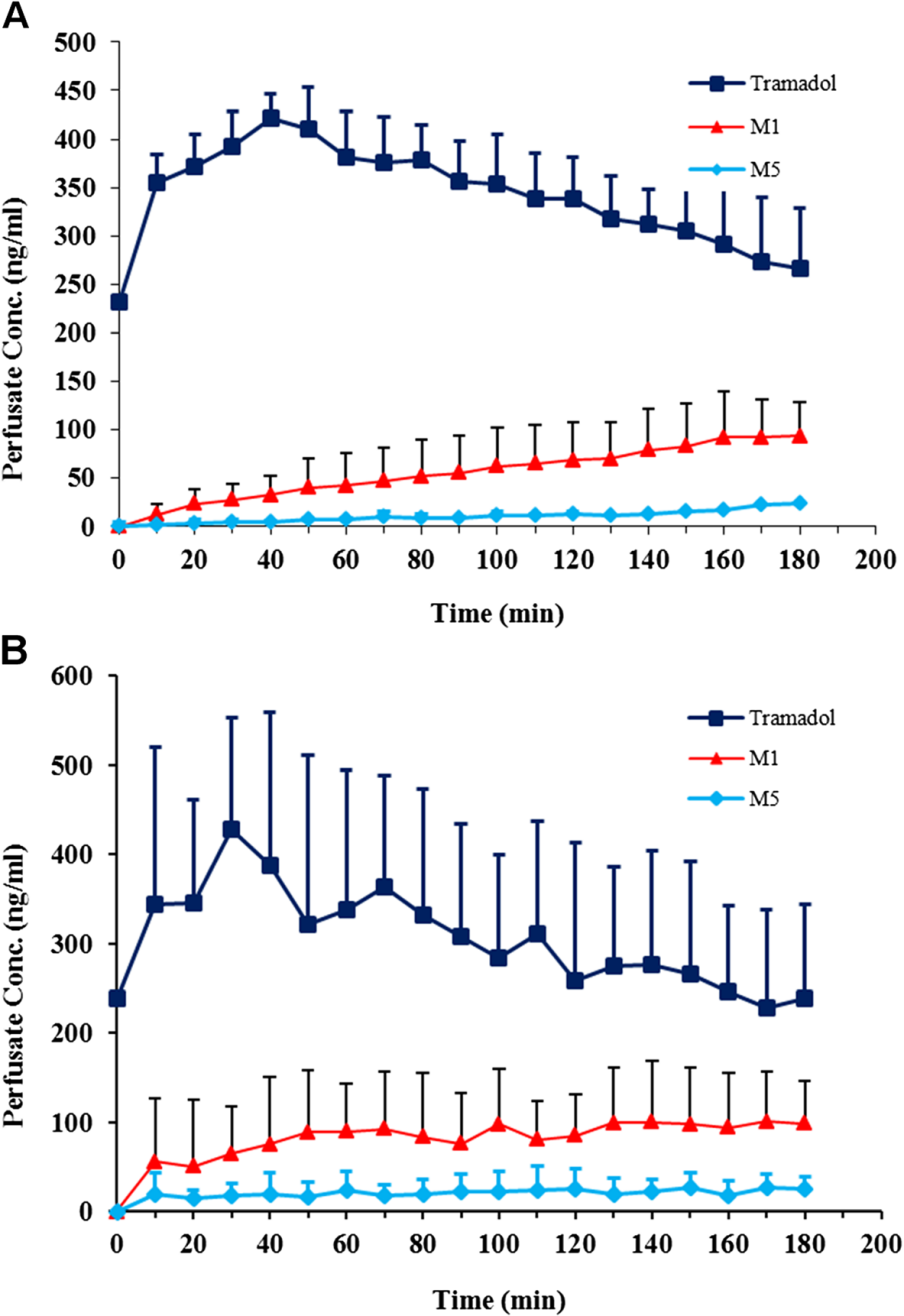
Perfusate concentration-time profile of tramadol, M1 and M5 in control (A) and diabetic (B) isolated rat liver (n = 6, data are presented as mean ± SE).

Six rats completed the study in each group. Similar to intact groups, the body weight and blood glucose levels between two groups were significantly different (P < 0.05). Perfusate sample concentrations of tramadol, M1 and M5 measured by HPLC in both groups. Similar to our previous study [20], M2 was not detected in isolated liver study samples. Figure 5 represents perfusate concentration-time profiles of tramadol, M1 and M5 in control and diabetic isolated livers respectively.

Similar to our previous study [20], M1 was the main metabolite formed in both diabetic and control livers and M2 metabolite was not seen (at least in concentrations higher than 2.5 ng/ml) in perfusion study. A comparison between concentration levels of M1 in diabetic and control livers showed that in contrast to intact animals, the metabolic ratios of M1 and M5 (M/T) are significantly higher in diabetic rats compared to those of control group (Figures 4 and 5). This higher metabolic ratio in diabetic livers is the consequence of higher metabolite (M1 and M5) production, a result that could not easily be concluded in intact animal data. So it could be fulfilled that higher metabolic ratios in intact control rats in comparison to intact diabetic rats could be resulted from lower plasma levels of tramadol itself in control group (instead of higher metabolite production) which most possibly has been resulted from higher volume of distribution and lower protein binding of tramadol. To confirm the exact role of distribution volume and even protein binding on tramadol concentration and their effects on metabolic ratios in control and diabetic rats, the PK study of tramadol after iv administration and calculation of exact volume of distribution and clearance (instead of apparent Vd and Cl) is recommended.

## Conclusion

In conclusion, the results of this study show that the pharmacokinetic of tramadol and its three metabolites has been influenced by diabetic condition. The higher concentration of M1 could be a result of induction of tramadol to M1 formation pathway, while higher concentrations of tramadol itself can be explained by its lower volume of distribution in diabetic rats.

## Competing interests

The authors declare that they have no competing interests.

## Authors’ contributions

M-RR, YHA , MA, BSH and HL conceived the study. HL, BSH, YHA and LH performed the experimental work. All authors were involved in data analysis and interpretation. HL, BSH and YHA prepared the manuscript. All authors read and approved the final version.
